# Large-scale integration of cancer microarray data identifies a robust common cancer signature

**DOI:** 10.1186/1471-2105-8-275

**Published:** 2007-07-30

**Authors:** Lei Xu, Donald Geman, Raimond L Winslow

**Affiliations:** 1 The Institute for Computational Medicine and Center for Cardiovascular Bioinformatics and Modeling, Johns Hopkins University, Baltimore, MD 21218, USA; 2 Department of Applied Mathematics and Statistics and Center for Imaging Sciences, Johns Hopkins University, Baltimore, MD 21218, USA

## Abstract

**Background:**

There is a continuing need to develop molecular diagnostic tools which complement histopathologic examination to increase the accuracy of cancer diagnosis. DNA microarrays provide a means for measuring gene expression signatures which can then be used as components of genomic-based diagnostic tests to determine the presence of cancer.

**Results:**

In this study, we collect and integrate ~ 1500 microarray gene expression profiles from 26 published cancer data sets across 21 major human cancer types. We then apply a statistical method, referred to as the *T*op-*S*coring *P*air of *G*roups (TSPG) classifier, and a repeated random sampling strategy to the integrated training data sets and identify a common cancer signature consisting of 46 genes. These 46 genes are naturally divided into two distinct groups; those in one group are typically expressed less than those in the other group for cancer tissues. Given a new expression profile, the classifier discriminates cancer from normal tissues by ranking the expression values of the 46 genes in the cancer signature and comparing the average ranks of the two groups. This signature is then validated by applying this decision rule to independent test data.

**Conclusion:**

By combining the TSPG method and repeated random sampling, a robust common cancer signature has been identified from large-scale microarray data integration. Upon further validation, this signature may be useful as a robust and objective diagnostic test for cancer.

## Background

During the past century, the presence of cancer in tissues has been diagnosed on the basis of histopathology [1]. The major limitation of this approach is that it cannot achieve high accuracy of prediction in clinical practice. Therefore, there has been a persistent need to identify robust cancer signatures which could complement conventional histopathologic evaluation to increase the accuracy of cancer detection [2]. More recently, DNA microarrays have been developed as a means to simultaneously measure the transcript abundance (gene expression level) of mRNA for thousands of genes. This technology provides a potentially powerful tool for identifying molecular signatures capable of accurately detecting the presence of cancer.

Many studies have used DNA microarrays to identify cancer type-specific gene expression signatures which can discriminate certain types of cancer from normal tissues [3-15]. The diversity of these signatures makes it difficult to distinguish the genes that play a crucial role in oncogenic processes from those that are spuriously differentially expressed and therefore irrelevant to the oncogenic processes. Since all cancer cells share two common characteristics, uncontrollable growth and local tissue invasion or metastasis, it is of high importance to identify a universal cancer type-independent signature to better understand cancer pathogenesis and ultimately to improve therapeutics. After such a signature is identified, it could be used as a component of genomic-based clinical diagnostic tools for cancer patients to determine the presence of cancer cells in tissues.

Recently, several studies used meta-analysis methods to identify genes differentially expressed across multiple cancer types [16-18]. In the study of Rhodes *et al*. [17], 21 published cancer microarray data sets, spanning 12 distinct cancer types, were collected and analyzed in an effort to identify a cancer type-independent transcriptional signature of neoplastic transformation. A statistical meta-analysis method, termed comparative meta-profiling, was proposed to compare and assess the intersection of many cancer type-dependent signatures, the goal being to identify a common cancer meta-signature. The comparative meta-profiling method works as follows. First, an overexpression direction (e.g. cancer > normal) and significant threshold are set to define differential gene expression signatures from a set of cancer versus normal studies (one signature per study). Next, genes are sorted by the number of signatures in which they are present. Finally, a meta-signature is defined as those genes appearing in a given number of signatures [17], where the cutoff is determined by a random simulation. Rhodes *et al *used this method to identify a set of 67 genes that are universally activated in most cancer types relative to corresponding normal tissues.

**Figure 1:**
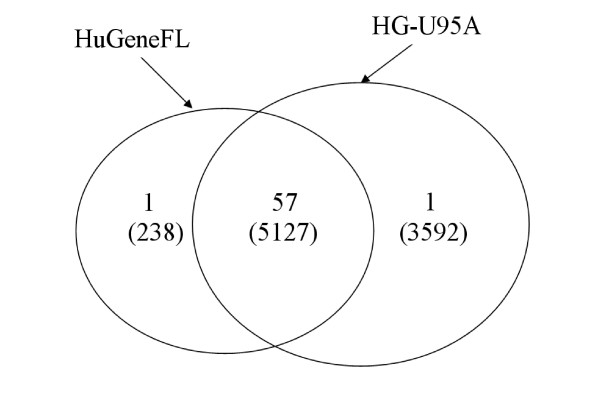
**Common genes overrepresented in the meta-signature**. The figure shows the relationship between the numbers of genes on two microarray platforms, HuGeneFL and HG-U95A, and the corresponding numbers of genes in the meta-signature of neoplastic transformation [17]. There are 5127 genes common to both platforms, 238 only on HuGeneFL and 3592 only on HG-U95A. The numbers without parentheses are the corresponding numbers of genes in the meta-signature.

One limitation of meta-analysis of microarray data is that small sample sizes typical of individual studies, coupled with variation due to differences in study protocols, inevitably degrades the results of meta-analysis. An additional and major limitation of the comparative meta-profiling method is that those genes which are common to the various array platforms used in these studies are highly overrepresented in the identified meta-signature. This way of defining a meta-signature by gene enrichment in signatures implies that many potentially informative genes which are not common to the various array platforms used in these studies may be overlooked due to the intrinsic properties of this method. As a specific example, the relationship between the numbers of total genes on two major Affymetrix microarray platforms used in the study of Rhodes *et al*. and the corresponding numbers of genes included in the reported common cancer meta-signature is shown in the Venn diagram of Figure 1. Among the 67 meta-signature genes, 59 genes are on one or both of these two microarray platforms and the other eight genes come from other microarray platforms. Almost all of the 59 meta-signature genes come from the set of 5127 genes which are common across the two microarray platforms employed in this study.

In our previous work, we proposed a novel, simple method, referred to as the top-scoring pair (TSP) classifier, to integrate inter-study microarray data and validated the method on prostate cancer data [14]. It has also been applied recently to differentiating between gastrointestinal stromal tumors and leiomyosarcomas [19]. The TSP method is based only on the ranks of the expression values within a profile and hence there is no need to perform data normalization and transformation before data integration because rankings are invariant to nearly all types of preprocessing. Further, the TSP method identifies only a pair of genes as a gene signature and the decision rule is based simply on comparing their expression values. This approach to data integration was validated on prostate cancer data [14], resulting in a powerful two-gene diagnostic classifier. Here, we extend this method to predict distant metastases in breast cancer, and attempt to overcome the limitations of previous study-specific methods and meta-analyses.

Here, we present a generalization of this method, termed top-scoring pair of groups (TSPG), which preserves the basic properties of the TSP classifier, namely invariance to normalization and comparison-based rules, but incorporates more genes into the decision-making. We apply this generalization to identify a cancer type-independent signature by integrating microarray data from different cancer studies across almost all major human cancer types. This overcomes the limitations of previous meta-analyses by integrating large-scale microarray data generated using the same microarray platform, resulting in one signature per platform (rather than per study) and effectively increasing sample size. Integrating microarray data from the same platform can guarantee that all the genes on the platform will be included in the analysis, therefore avoiding losing any potential signature genes. Moreover, unlike the approach in [17], which requires cross-validation for decision-making, and hence an ensemble of new samples for testing the predictive power of the common signature, the TSPG method provides a classifier in the standard sense of a prediction rule which can be applied to label a single new sample.

By combining the TSPG method and a repeated random sampling strategy, a common cancer signature, which consists of 46 distinct genes, is identified from the integrated microarray data. The TSPG classifier, which discriminates cancer from normal samples, is built from the signature and validated on both the training data and independent cancer microarray data. Upon further validation on large-scale independent data, the signature may be used to develop a novel cancer diagnostic tool and provide new insights regarding the mechanism of cancer.

## Results

### Data collection

Microarray data sets were obtained from public gene expression data repositories, including Gene Expression Omnibus [20], Oncomine [21] and supporting web sites identified from the published literature. In particular, we focused on analysis of human cancer gene expression data obtained using the Affymetrix HuGeneFL, HG-U95A and HG-U133A microarray platforms. Of these collected data, 26 individual data sets generated from HuGeneFL arrays (Table 1) and HG-U95A arrays (Table 2) were used as training data and six individual data sets from HG-U133A arrays (Table 3) were used as independent test data. In total, 1593 microarray experiments from 32 independent studies across almost all human cancer types were used in this analysis. Studies are named using the same convention as in [17]: FirstAuthor_Tissue (e.g. Beer_Lung).

**Table 1:** Microarray data from Affymetrix HuGeneFL arrays

Study	Class1	Size	Class2	Size
Beer_Lung ^[25]^	Normal Lung	10	Lung Adenoarcinoma	86
Dyrskjot_Bladder ^[26]^	Normal Bladder	4	Bladder Cancer	40
Hippo_Gastric ^[27]^	Normal Gastric Tissues	8	Gastric Cancer	22
Hsiao_Normal ^[28]^	Normal Tisues	59		
Lancaster_Ovarian ^[29]^	Normal Ovary	3	Ovarian Adenocarcinoma	31
Logsdon_Pancreas ^[30]^	Normal Pancreas	5	Pancreatic Adenocarcinoma	10
Pomeroy_Brain ^[31]^	Normal Cerebellum	4	Atypical Teratoid/Rhabdoid Tumors	10
			Primitive neuroectodermal Tumors	8
			Malignant Gliomas	10
			Medulloblastoma	10
Quade_Myometrium ^[32]^	Normal Myometrium	4	Leiomyosarcoma	14
Ramaswamy_Multi ^[33]^	Normal Prostate	9	Prostate Cancer	10
	Normal Uterus	6	Uterine Cancer	10
	Normal Whole brain	8	Glioblastoma/Medulloblastoma	20
	Normal Breast	5	Breast Adenocarcinoma	11
	Normal Lung	7	Lung Adenoarcinoma	11
	Nromal Colon	11	Colorectal	11
	Normal Germinal Center	6	Lymphoma	22
	Normal Bladder	7	Bladder Cancer	11
			Melanoma	10
	Peripheral Blood	5	Leukemia	30
	Normal Kidney	12	Renal Cell Carcinoma	11
	Normal Pancreas	10	Pancreatic Adenocarcinoma	11
	Normal Ovary	4	Ovarian Carcinoma	11
			Mesothelioma	11
Rickman_Brain ^[34]^	Normal Temporal Lobe	6	Glioma	45
Welsh_Ovarian ^[35]^	Normal Ovary	4	Ovarian Carcinoma	22
Zhan_Myeloma ^[36]^	Normal Plasma Cell- Bone Marrow	30	Multiple Myeloma	74

Total	Normal Tissues	227	Cancer Tissues	572

**Table 2:** Microarray data from Affymetrix HG-U95A arrays

Study	Class1	Size	Class2	Size
Bhattacharjee_Lung ^[37]^	Normal Lung	17	Small Cell Lung Carcinoma	6
			Lung Carcinoid	20
			Squamous Cell Lung Carcinoma	21
Cromer_Head-Neck ^[38]^	Normal Uvula	4	Head-Neck Squamous Cell Carcinoma	34
Dehan_Lung ^[39]^	Normal Lung	9	Lung Adenocarcinoma	7
			Lung Squamous Cell Carcinoma	17
			Lung Adenosquamous	1
Frierson_Salivary ^[40]^	Normal Salivary Gland	6	Salivary Carcinoma	16
Giordano_Adrenal ^[41]^	Normal Adrenal Cortex	3	Adrenocortical Carcinoma	11
Gutmann_Brain ^[42]^	Normal White Matter	3	Pilocytic Astrocytoma	8
Huang_Thyroid ^[43]^	Normal Thyroid	8	Thyroid Carcinoma	8
Shai_Brain ^[44]^	White Matter	7	Glioblastoma Multiforme	35
Stearman_Lung ^[45]^	Normal Lung	19	Lung Tumor	20
Su_Multi ^[46]^	Normal Tissues	63	Tumor Tissues	18
Su_Tumors ^[5]^			Prostate Cancer	24
			Bladder/Ureter	8
			Breast	21
			Colorectal	21
			Gastroesophagus	11
			Kidney	10
			Liver	6
			Ovary	23
			Pancreas	6
			Lung Adenocarcinoma	12
			Lung Squamous Cell Carcinoma	12
Welle_Normal ^[47]^	Normal Muscle	12		
Yanai_Normal ^[48]^	Normal Tissues	24		
Yu_Prostate ^[49]^	Normal Prostae	16	Primary Prosate Carcinoma	35

Total	Normal Tissues	191	Cancer Tissues	411

**Table 3:** Microarray data from Affymetrix HG-U133A arrays

Study	Class1	Size	Class2	Size
Gordon_Lung ^[50]^	Normal Lung	4	Malignant Pleural Mesothelioma	40
	Normal Pleura	5		
Hoffman_Myometrium ^[51]^	Normal Myometrium	5	Uterine Leiomyomas	5
Lenburg_Kidney ^[52]^	Normal Kidney Tissue	5	Renal Cell Carcinoma	12
Talantov_Skin ^[53]^	Normal Skin	7	Melanoma	45
Wachi_Lung ^[54]^	Normal Lung	5	Squamous Lung Cancer	5
Yoon_Soft_Tissue ^[55]^	Normal Soft Tissue	15	Soft Tissue Sarcoma	39

Total	Normal Tissues	46	Cancer Tissues	146

### Common cancer signature

We directly merge 12 (respectively, 14) cancer/normal microarray data sets generated from Affymetrix HuGeneFL (respectively, HG-U95A) (Table 1 and 2), using the common 7069 (respectively, 12532) probe sets among all these data sets to form an integrated training data set with 799 (respectively, 602) samples. These data sets span 21 tissue types including lung, breast, bladder, ovarian, pancreas, brain, prostate, uterus, colon, blood, kidney, uvula, salivary gland, thyroid gland, liver, skin, gastric tissue, myometrium, bone marrow, adrenal cortex and gastroesophagus. For each of the two integrated data sets, we randomly select *S*% = 90% of the total samples from the integrated data set and then apply the TSPG algorithm to the selected data to construct two groups, *G*_1 _and *G*_2_, of genes. After the experiment is repeated 1000 times, the appearance frequency for each gene which is present in any of the 1000 *G*_1_'s (respectively, *G*_2_'s) is calculated. For the default frequency threshold *F *= 80%, the appearance frequency of 24 genes (13 in *G*_1 _and 11 in *G*_2_) from the HuGeneFL integrated data set and 25 genes (12 in *G*_1 _and 13 in *G*_2_) from the HG-U95A integrated data set exceeds *F *(Table 4). There are three genes (CLEC3B, COX7A1 and KIAA0101) which are selected from both integrated data sets. Therefore, a common cancer signature, which consists of the 46 genes (24 in *G*_1 _and 22 in *G*_2_) obtained from the two integrated data sets, is identified from the integrated microarray data. For *F *= 90% and 70% the common cancer signatures consist of slightly different genes. (For *F *= 90%, 39 out of the above 46 genes appear and for *F *= 70% 10 more genes are added to the 46 genes.) For the rest of this paper, we will focus on the 46-gene common cancer signature corresponding to *F *= 80%.

**Table 4:** Common cancer signature genes

Microarray Platform	*G*_1_		*G*_2_	
	
	Gene Symbol	Probe Set ID	Gene Symbol	Probe Set ID
HuGeneFL	BOP1	D50914_at	COX7A1	M83186_at
	PON2	L48513_at	CXCL12	U19495_s_at
	NME1*	X17620_at	ALDH1A1	M31994_at
	CKS2*	X54942_at	SELP	M25322_at
	CCT3	X74801_at	CD36	Z32765_at
	KIAA0101*	D14657_at	CSRP1	M76378_at
	FOXM1	U74612_at	C9orf61	L27479_at
	MAP3K11	L32976_at	MYH11	AF001548_rna1_at
	RAB13	X75593_at	LTC4S	U50136_rna1_at
	ARPC1B	AF006084_at	DEFA4	X65977_at
	HMGA1	L17131_rna1_at	CLEC3B	X64559_at
	TYMS	D00596_at		
	DNMT1	X63692_at		

HG-U95A	SOX4*	33131_at	TEK	1596_g_at
	C7orf24	41696_at	FXYD1	32109_at
	POSTN	1451_s_at	ABCA8	35717_at
	BAZ1B	32261_at	CLEC3B	36569_at
	KIAA0101*	38116_at	CBX7	36894_at
	RECQL	34684_at	TNXA///TNXB	38508_s_at
	FAT	40454_at	SH3BP5	38968_at
	SIPA1L3	37831_at	CA4	40739_at
	MARCKSL1	36174_at	FBXO9	38990_at
	CKAP4	32529_at	COX7A1	39031_at
	KIF14	34563_at	GABARAPL1	35785_at
	SUB1	36171_at	ADH1B	35730_at
			PTGDS	216_at

* These genes were also identified as common cancer signature genes in Rhode *et al*. [17].

### Validation of the common cancer signature on the training data

**Figure 2:**
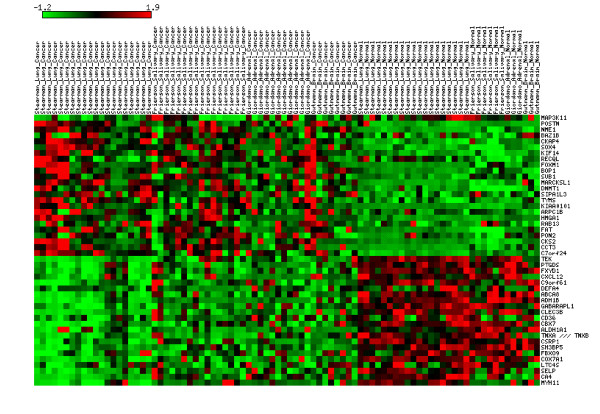
**Common cancer signature which can discriminate cancer from normal samples**. Some of the training data (Stearman_Lung, Frierson_Salivary, Giordano_Adrenal and Gutmann_Brain) is used to illustrate the gene expression values of the signature genes in the figure. The heatmap is generated by the matrix2png software [24]. For each data set, the expression value for each gene is normalized across the samples to zero mean and one standard deviation (SD) for visualization purposes. Genes with expression levels greater than the mean are colored in red and those below the mean are colored in green. The scale indicates the number of SDs above or below the mean.

To validate the reliability and robustness of the common cancer signature, the TSPG classifier, which predicts cancer vs. normal status, is built based on all the signature genes, with 24 genes in *G*_1 _and 22 genes in *G*_2 _as indicated above. The classification rule for the TSPG classifier is that if the average relative rank of the genes in *G*_1 _is less than that of the genes in *G*_2_, a test sample is classified as normal; otherwise it is classified as cancer. The expression values of the 46 signature genes are illustrated in Figure 2 using some of the training data, including Stearman_Lung, Frierson_Salivary, Giordano_Adrenal and Gutmann_Brain data sets. Distinct patterns of expression values of the genes in *G*_1 _and *G*_2 _can be observed for normal and cancer samples. The classifier is then used to assess the prediction accuracy of the signature on the training data sets spanning a wide range of cancer types. For the 26 individual data sets which have been integrated and used to identify the signature, the classification accuracy and the *p*-values of the Fisher's exact test are shown in Table 5. The classifier achieves high accuracy (> 85%) on 19 of 26 data sets and the overall accuracy is about 86%. From the *p*-values of the Fisher's exact test, we learn that the classification is significant (*p*-value < 0.03) on 18 of 22 data sets. There is no *p*-value available for four data sets which only have samples from one class. The classifier is both significant (*p*-value < 0.03) and accurate (> 85%) on 14 of 22 data sets. The results suggest that we have identified a common cancer signature for most, if not all, human cancer types.

**Table 5:** Class prediction of the common signature on training data

Microarray Platform	Study	Number of Normal Samples	Number of Cancer Samples	Accuracy (%)	*P*-value
HuGeneFL	Beer_Lung	10	86	95.8	8.87E-11
	Dyrskjot_Bladder	4	40	95.5	1.18E-03
	Hippo_Gastric	8	22	76.7	2.67E-01
	Hsiao_Normal	59	0	91.5	N/A*
	Lancaster_Ovarian	3	31	91.2	1.00
	Logsdon_Pancreas	5	10	100	3.33E-04
	Pomeroy_Brain	4	38	97.6	3.48E-04
	Quade_Myometrium	4	14	77.8	2.29E-02
	Ramaswamy_Multi	90	190	77.9	1.28E-20
	Rickman_Brain	6	45	94.1	2.87E-04
	Welsh_Ovarian	4	22	100	6.69E-05
	Zhan_Myeloma	30	74	72.1	2.88E-01
	**Overall (HuGeneFL)**	**227**	**572**	**85.0**	**6.70E-68**

HG-U95A	Bhattacharjee_Lung	17	47	90.6	6.34E-10
	Cromer_Head-Neck	4	34	97.4	4.74E-04
	Dehan_Lung	9	25	85.3	3.82E-05
	Frierson_Salivary	6	16	95.5	9.38E-05
	Giordano_Adrenal	3	11	92.9	1.10E-02
	Gutmann_Brain	3	8	100	6.06E-03
	Huang_Thyroid	8	8	75	5.59E-02
	Shai_Brain	7	35	85.7	6.36E-05
	Stearman_Lung	19	20	89.7	1.28E-07
	Su_Multi	63	18	81.5	1.15E-05
	Su_Tumors	0	154	93.5	N/A
	Welle_Normal	12	0	100	N/A
	Yanai_Normal	24	0	91.7	N/A
	Yu_Prostate	16	35	54.9	1.83E-02
	**Overall (HG-U95A)**	**191**	**411**	**87.0**	**3.65E-77**

* For the data sets with samples from only one class, no *p*-value is available.

### Validation of the common cancer signature on independent test data

To further validate the generality and robustness of the common cancer signature, the TSPG classifier built based on the signature is tested on six independent test data sets generated from a different generation of Affymetrix microarray platforms, HG-U133A (Table 3). The six independent data sets represent six different human cancer types, one of which is not represented in the training data sets (Yoon_Soft_Tissue). The classification accuracy and statistical significance (i.e. *p*-value of the Fisher's exact test) of the common cancer signature are listed in Table 6. The signature significantly (*p*-value < 0.005) discriminates cancer from normal samples with very high accuracy (> 95%) on four of the six data sets, including the new cancer type data set. On the other two data sets, the signature achieves much higher accuracy (> 75%) than that of coin-flipping but only marginally significant (*p*-value = 0.083 and 0.107). The independent test results have validated that the signature is common to a wide range of cancer types and may be used to detect the presence of cancer cells in tissues.

**Table 6:** Validation of the common signature on independent HG-U133A data

Study	Number of Normal Samples	Number of Cancer Samples	Accuracy (%)	*P*-value
Gordon_Lung	9	40	95.9	1.75E-07
Hoffman_Myometrium	5	5	80.0	8.33E-02
Lenburg_Kidney	5	12	76.5	1.07E-01
Talantov_Skin	7	45	98.1	3.44E-07
Wachi_Lung	5	5	100	3.97E-03
Yoon_Soft_Tissue	15	39	96.3	6.76E-11

**Overall**	**46**	**146**	**94.3**	**9.74E-30**

### Comparison with the Rhodes signature

We compare our common cancer signature to the one for meta-signature of neoplastic transformation reported in [17]. It is not surprising that there is a small overlap between the two signatures with four common genes (see Table 4). On the one hand, this implies that both studies have identified some molecular features common to all cancer types. On the other hand, the difference between the two signatures may result from the two major differences between the two methods. First, the comparative meta-profiling method overlooks a large number of potential signature genes due to its intrinsic properties whereas our method includes all possible genes in the analysis. Second, the analysis in [17] focuses on differentially expressed genes; more specifically, it chooses genes which are over-expressed in one direction (cancer > normal). In contrast, our selection criterion is not based on differential expression, but rather on finding pairs of genes whose expression values typically invert from cancer to normal tissues. One consequence is that our signature includes genes over-expressed in both directions, as illustrated in Figure 2, as well as genes which are not differentially expressed.

We then compare the classification performance of the two signatures on the same independent test data. Results are reported in Table 7. In this way the two signatures and corresponding cancer prediction rules are evaluated on completely independent data not involved in learning either signature. Significantly, the approach in Rhodes [17] does not provide a well-defined classifier corresponding to the signature; important aspects of the classifier must be learned on the test data, producing an upwardly-biased estimate of classification accuracy. (A more complete explanation appears in "Methods.") Nonetheless, the TSPG classifier based on our common signature out-performs the classifier based on the Rhode signature [17] on three of the six independent test data sets and achieves the same accuracy on the other three data sets. Unlike the Rhodes classifier, the TSPG classifier is completely determined by the signature itself and was applied sample-by-sample to the test data; no learning on the test data was involved.

**Table 7:** Comparison with the Rhodes signature on the same independent data

Study	Rhodes Signature		Our Signature	
	
	Accuracy (%)	*P*-value	Accuracy (%)	*P*-value
Gordon_Lung	91.8	3.48E-07	95.9	1.75E-07
Hoffman_Myometrium	80.0	2.06E-01	80.0	8.33E-02
Lenburg_Kidney	76.5	1.01E-01	76.5	1.07E-01
Talantov_Skin	94.2	8.97E-07	98.1	3.44E-07
Wachi_Lung	100	3.97E-03	100	3.97E-03
Yoon_Soft_Tissue	85.2	5.67E-8	96.3	6.76E-11

**Overall**	**89.1**	**9.28E-26**	**94.3**	**9.74E-30**

## Discussion

The advent of DNA microarray has had a tremendous impact on cancer research. This technology provides a novel molecular tool, complementary to histopathologic examination, to assess the presence of cancer cells in patient tissues. The rapid accumulation of cancer microarray data makes it possible to integrate a large amount of microarray gene expression data across a wide range of cancer types to identify a universal cancer signature to detect cancer cells, regardless of the tissue from which the cancer is derived. In this study, by integrating microarray data and applying the TSPG method combined with repeated random sampling, we have identified a robust cancer gene expression signature common to almost all major human cancer types. The discriminative power of the signature has been validated on both data sets involved in identifying the signature and independent test data sets. The TSPG classifier built from the signature, which simply compares the average relative ranks of two groups of genes, achieves high accuracy on most of the training and test data sets with statistical significance. Although the signature has the potential to be developed as a robust and objective clinical diagnostic test for cancer, larger number of samples will be required to further refine and validate it.

An intriguing advantage of inter-study cancer microarray data integration is that it increases the statistical power to capture essential, cancer type-independent gene expression features, which might be masked by specific features of individual cancer types and small sample sizes of individual data sets. In this sense, the signature is reliable and robust to variations in individual cancer data sets. The universal cancer signature described here may play a crucial role in oncogenic processes and be used to improve our understanding of cancer pathogenesis and ultimately design improved anticancer treatments. It also suggests the possible existence of therapeutic targets common to different cancer types.

We have observed that the signature seems to perform better for certain tissue types, such as lung, skin, and soft tissue cancer. There are many possible reasons to cause the different performance of the signature on different cancer types. These reasons may include differences in sample composition and preparation, experimental protocols, RNA quality, proportion of cancer cells in a tissue, and microarray quality. In addition, some cancer types might be easier to detect than other cancer types.

It is not surprising that many of the signature genes (BOP1, KIAA0101, CCT3, ARPC1B, CKAP4, ALDH1A1, CD36, CLEC3B, TEK, CBX7) have been reported to be associated with specific types of cancer in the literature and some other genes (NME1, TYMS, POSTN, FOXM1, HMGA1, DNMT1, KIF14, CXCL12, SELP) have been previously found to be associated with a variety of distinct human cancer types. As defined by Gene Ontology Consortium, the common signature genes are involved in cell cycle (MAP3K11, NME1, CKS2, MYH11), regulation of transcription (BAZ1B, SOX4, FOXM1, SUB1, CBX7, DNMT1, HMGA1, RAB13), DNA metabolism (RECQL, CBX7, DNMT1, TYMS, HMGA1), cellular biosynthesis (NME1, PTGDS, KIF14, TYMS, LTC4S), cellular protein metabolism (MAP3K11, TEK, KIF14, FBXO9, HMGA1, CCT3) and other important biological processes, such as cell organization and cell adhesion. These findings are consistent with the fact that all cancer types share the common features of uncontrollable cell growth and local tissue invasion, and therefore the genes that are essential to these cellular processes are possible signature genes among almost all cancer types.

One limitation of our proposed method for microarray data integration is that it can only directly integrate microarray data generated from the same standard microarray platforms. Even with this limitation, we still obtain a large number of samples (> 500) on each of the two microarray platforms used in this study. With the rapid increase of available microarray data and the standardization of microarray technologies, the mass of microarray data generated from the same platforms will continue to grow, which will make our method become increasingly useful.

It is quite interesting that a similar study on common cancer biomarkers was published very recently [22]. The uniqueness of the study is that the researchers have generated microarray data across various cancer types using the same spotted cDNA microarray, and therefore no data integration is needed. By applying a gene pairing method to a training set with 201 samples of various normal and cancer tissues, a subset of 14 genes identified as common cancer biomarkers with high predictive power (87%) in segregating cancer from normal samples. Two of the 14 genes, PON2 and SOX4, have also been identified in our common cancer signature. The major limitation of the study is that the cancer samples are dominated by only a few cancer types (colon, melanoma, ovarian and esophageal cancers). Therefore, the biomarkers identified in the study may not really be common to a broad range of cancer types. In our study, motivated by the work of Rhodes *et al*., we collected a broader range of microarray gene expression data for about 20 cancer types and each of them is reasonably represented in the training data sets. The signature identified in our study has been validated on independent data sets of various cancer types, including one cancer type which is not represented in the training data sets. 

Finally, we have also compared our classifier with the method in Rhodes *et al*. on the same test sets; in this way the two signatures and corresponding cancer prediction rules are evaluated on completely independent data not involved in learning either signature. In classifying a test sample, the Rhodes classifier must use all the other samples in the same test set to compute class averages; hence cross-validation is necessary. In contrast, our classifier operates in the conventional way, classifying each sample independently of all others. Despite this source of bias in the Rhodes classifier, our method still achieves higher overall accuracy.

## Conclusion

In conclusion, by combining large-scale microarray data, a robust common cancer signature has been identified. Upon more large-scale validation, it could be developed as a component of genomic-based clinical diagnostic tools for cancer patients. Further studies of the signature might also improve our understanding of cancer and identify new drug targets.

## Methods

### TSPG classifier

Recently, our group has developed a family of statistical molecular classification methods, referred to as the TSP and the *k*-TSP classifiers [13,15], and applied the TSP classifier to microarray data integration [14]. These methods only use the ranks of gene expression values within each profile and achieve impressive results in both molecular classification and microarray data integration [13-15]. An important feature of rank-based methods is that they are invariant to monotonic transformations of the expression data within an array, such as those used in most data normalization methods. This property makes these methods useful for combining inter-study microarray data without the need to perform data normalization and transformation. Here, we present a similar but more robust method, termed the TSPG classifier, in order to identify a gene expression signature common to a wide range of cancer types by integrating large-scale cancer microarray data.

The development of the TSPG method is motivated by following considerations. It is known that many genes are involved in the oncogenic processes; therefore, in order to better understand cancer pathogenesis, we need to identify a common cancer signature which consists of more than just a few genes. The TSP method previously used only identifies a pair of signature genes, say *i *and *j*, which obviously cannot constitute an effective common cancer signature. The basic idea it to replace *i *and *j *by two groups *G*_1 _and *G*_2 _of genes, but preserve the property of basing prediction on expression comparisons as well as the scoring mechanism. Instead of simply comparing the ranks of the expression values of genes *i *and *j*, we rank all the genes in the two groups *G*_1 _and *G*_2 _and compare the average ranks *G*_1 _and *G*_2_. When each group consists of a single gene, the TSPG method reduces to TSP.

Another motivation for developing the TSPG method is related to the observation that, in some cases, one gene may pair with different genes to form a top-scoring pair when the training data is perturbed by adding or deleting a few samples. This may imply that a gene consistently appearing in top-scoring pairs may be closely correlated to cancer whereas the other genes occasionally paired with it might be irrelevant to cancer. We want to keep those genes which may play a crucial role in oncogenic processes in the common cancer signature and eliminate those genes which may be irrelevant to cancer. When combined with repeated random sampling, the TSPG method provides the flexibility to keep one gene of a TSP in a signature while excluding the other one from it.

The TSPG classifier is defined as follows. Assume the training microarray data are represented by a *P *× *N *matrix ***X ***= [*X*_*pn*_], *p *= 1, 2, ..., *P *and *n *= 1, 2, ..., *N*, where *P *is the number of genes in each profile and *N *is the number of samples (i.e. profiles). Each column *n *represents a gene expression profile of *P *genes with a class label *Y*_*n *_= 1 (normal) or 2 (cancer) for the two-class problem (normal vs. cancer) in this study. Among the *N *samples, there are *N*_1 _(respectively, *N*_2_) samples labeled as class 1 (respectively, class 2) with *N *= *N*_1 _+ *N*_2_. For each pair of genes (*i*, *j*), where *i*, *j *= 1, 2, ..., *P*, *i *≠ *j*, we define a score as

*Δ*_*ij *_= |*P*(*X*_*i *_<*X*_*j *_| *Y *= 1) - *P*(*X*_*i *_<*X*_*j *_| *Y *= 2)|

and estimate the score based on the training set ***X ***by

Δij≈|Nij(1)N1−Nij(2)N2|
 MathType@MTEF@5@5@+=feaafiart1ev1aaatCvAUfKttLearuWrP9MDH5MBPbIqV92AaeXatLxBI9gBaebbnrfifHhDYfgasaacH8akY=wiFfYdH8Gipec8Eeeu0xXdbba9frFj0=OqFfea0dXdd9vqai=hGuQ8kuc9pgc9s8qqaq=dirpe0xb9q8qiLsFr0=vr0=vr0dc8meaabaqaciaacaGaaeqabaqabeGadaaakeaacqqHuoardaWgaaWcbaGaemyAaKMaemOAaOgabeaakiabgIKi7oaaemaabaWaaSaaaeaacqWGobGtdaqhaaWcbaGaemyAaKMaemOAaOgabaGaeiikaGIaeGymaeJaeiykaKcaaaGcbaGaemOta40aaSbaaSqaaiabigdaXaqabaaaaOGaeyOeI0YaaSaaaeaacqWGobGtdaqhaaWcbaGaemyAaKMaemOAaOgabaGaeiikaGIaeGOmaiJaeiykaKcaaaGcbaGaemOta40aaSbaaSqaaiabikdaYaqabaaaaaGccaGLhWUaayjcSdaaaa@48E6@

where

Nij(k)=|{n:1≤n≤N,Xin<Xjn,Yn=k}|,k=1,2.
 MathType@MTEF@5@5@+=feaafiart1ev1aaatCvAUfKttLearuWrP9MDH5MBPbIqV92AaeXatLxBI9gBaebbnrfifHhDYfgasaacH8akY=wiFfYdH8Gipec8Eeeu0xXdbba9frFj0=OqFfea0dXdd9vqai=hGuQ8kuc9pgc9s8qqaq=dirpe0xb9q8qiLsFr0=vr0=vr0dc8meaabaqaciaacaGaaeqabaqabeGadaaakeaacqWGobGtdaqhaaWcbaGaemyAaKMaemOAaOgabaGaeiikaGIaem4AaSMaeiykaKcaaOGaeyypa0ZaaqWaaeaacqGG7bWEcqWGUbGBcqGG6aGocqaIXaqmcqGHKjYOcqWGUbGBcqGHKjYOcqWGobGtcqGGSaalcqWGybawdaWgaaWcbaGaemyAaKMaemOBa4gabeaakiabgYda8iabdIfaynaaBaaaleaacqWGQbGAcqWGUbGBaeqaaOGaeiilaWIaemywaK1aaSbaaSqaaiabd6gaUbqabaGccqGH9aqpcqWGRbWAcqGG9bqFaiaawEa7caGLiWoacqGGSaaliiaacqWFGaaicqWFGaaicqWGRbWAcqGH9aqpcqaIXaqmcqGGSaalcqaIYaGmcqGGUaGlaaa@5D1C@

For those pairs that achieve an identical score, we use a secondary score, called the rank-score, to break the tie [14,15]. The rank-score takes into account the extent to which a gene pair inverts from one class to the other. For each pair (*i*, *j*), the rank-score, denoted by *δ*_*ij*_, is defined as

δij=|1N1∑n∈{m:1≤m≤N,Ym=1}(Rin−Rjn)−1N2∑n∈{m:1≤m≤N,Ym=2}(Rin−Rjn)|.
 MathType@MTEF@5@5@+=feaafiart1ev1aaatCvAUfKttLearuWrP9MDH5MBPbIqV92AaeXatLxBI9gBaebbnrfifHhDYfgasaacH8akY=wiFfYdH8Gipec8Eeeu0xXdbba9frFj0=OqFfea0dXdd9vqai=hGuQ8kuc9pgc9s8qqaq=dirpe0xb9q8qiLsFr0=vr0=vr0dc8meaabaqaciaacaGaaeqabaqabeGadaaakeaaiiGacqWF0oazdaWgaaWcbaGaemyAaKMaemOAaOgabeaakiabg2da9maaemaabaWaaSaaaeaacqaIXaqmaeaacqWGobGtdaWgaaWcbaGaeGymaedabeaaaaGcdaaeqbqaaiabcIcaOiabdkfasnaaBaaaleaacqWGPbqAcqWGUbGBaeqaaOGaeyOeI0IaemOuai1aaSbaaSqaaiabdQgaQjabd6gaUbqabaGccqGGPaqkaSqaaiabd6gaUjabgIGiolabcUha7jabd2gaTjabcQda6iabigdaXiabgsMiJkabd2gaTjabgsMiJkabd6eaojabcYcaSiabdMfaznaaBaaameaacqWGTbqBaeqaaSGaeyypa0JaeGymaeJaeiyFa0habeqdcqGHris5aOGaeyOeI0YaaSaaaeaacqaIXaqmaeaacqWGobGtdaWgaaWcbaGaeGOmaidabeaaaaGcdaaeqbqaaiabcIcaOiabdkfasnaaBaaaleaacqWGPbqAcqWGUbGBaeqaaOGaeyOeI0IaemOuai1aaSbaaSqaaiabdQgaQjabd6gaUbqabaGccqGGPaqkaSqaaiabd6gaUjabgIGiolabcUha7jabd2gaTjabcQda6iabigdaXiabgsMiJkabd2gaTjabgsMiJkabd6eaojabcYcaSiabdMfaznaaBaaameaacqWGTbqBaeqaaSGaeyypa0JaeGOmaiJaeiyFa0habeqdcqGHris5aaGccaGLhWUaayjcSdGaeiOla4caaa@81AD@

Here *R*_*in *_is the rank of the expression value of gene *i *within the *n*-th profile in ascending order.

The TSPG algorithm constructs two disjoint groups (or sets) of genes, *G*_1 _and *G*_2_, with |*G*_1_|*+*|*G*_2_| <<*P*, where |*G*_*l*_| denotes the number of genes in group *G*_*l*_, *l *= 1,2. The classifier makes a prediction based on the average relative ranks of the genes in the two groups. The decision rule of the classifier is based on all the genes in *G*_1 _and *G*_2_. Let *W*_*i*_, *i *∈ *G *= *G*_1 _∪ *G*_2_, be the relative ranks of the genes within *G *in ascending order and let *E *denote the event that the average relative rank of the genes in *G*_1 _is less than that of the genes in *G*_2_, that is,

E={1|G1|∑i∈G1Wi<1|G2|∑j∈G2Wj}.
 MathType@MTEF@5@5@+=feaafiart1ev1aaatCvAUfKttLearuWrP9MDH5MBPbIqV92AaeXatLxBI9gBaebbnrfifHhDYfgasaacH8akY=wiFfYdH8Gipec8Eeeu0xXdbba9frFj0=OqFfea0dXdd9vqai=hGuQ8kuc9pgc9s8qqaq=dirpe0xb9q8qiLsFr0=vr0=vr0dc8meaabaqaciaacaGaaeqabaqabeGadaaakeaacqWGfbqrcqGH9aqpdaGadaqaamaalaaabaGaeGymaedabaWaaqWaaeaacqWGhbWrdaWgaaWcbaGaeGymaedabeaaaOGaay5bSlaawIa7aaaadaaeqbqaaiabdEfaxnaaBaaaleaacqWGPbqAaeqaaaqaaiabdMgaPjabgIGiolabdEeahnaaBaaameaacqaIXaqmaeqaaaWcbeqdcqGHris5aOGaeyipaWZaaSaaaeaacqaIXaqmaeaadaabdaqaaiabdEeahnaaBaaaleaacqaIYaGmaeqaaaGccaGLhWUaayjcSdaaamaaqafabaGaem4vaC1aaSbaaSqaaiabdQgaQbqabaaabaGaemOAaOMaeyicI4Saem4raC0aaSbaaWqaaiabikdaYaqabaaaleqaniabggHiLdaakiaawUhacaGL9baacqGGUaGlaaa@53A0@

The decision rule of the TSPG classifier is then very simple: Given a test sample ***X ***= [*X*_1_, ..., *X*_*P*_], if we observe that event *E*, then the classifier selects class 1; otherwise, it selects class 2. Notice the decision rule is based only on the relative ordering among the transcript abundances for the genes in *G*.

Ideally, the two groups of genes in the TSPG algorithm would be determined by maximizing a score analogous to (1), but with the event {*X*_*i *_<*X*_*j*_} replaced by *E*. Needless to say, this is an intractable optimization problem as it would involve optimizing over all pairs of subsets of genes. Instead, we do something sub-optimal but simple: we construct the groups directly from the *k *top-scoring pairs of genes, where we fix *k *= 20; no other values of *k *were attempted, although performance could likely be improved by choosing *k *based on cross-validation. Specifically, we first form a list of gene pairs, sorted from the largest to the smallest according to their scores Δ_*ij*_, with ties (if there are any) broken by using the rank-score *δ*_*ij*_. Next, the *k *disjoint top-scoring pairs are selected from the list. (In the *k*-TSP classifier [15], a decision is made by simple voting among the *k *top-scoring pairs.) Finally, we assign each of these selected genes to either *G*_1 _or *G*_2_. For each pair (*i*, *j*) among the *k *selected pairs, if Nij(2)/N2<Nij(1)/N1
 MathType@MTEF@5@5@+=feaafiart1ev1aaatCvAUfKttLearuWrP9MDH5MBPbIqV92AaeXatLxBI9gBaebbnrfifHhDYfgasaacH8akY=wiFfYdH8Gipec8Eeeu0xXdbba9frFj0=OqFfea0dXdd9vqai=hGuQ8kuc9pgc9s8qqaq=dirpe0xb9q8qiLsFr0=vr0=vr0dc8meaabaqaciaacaGaaeqabaqabeGadaaakeaacqWGobGtdaqhaaWcbaGaemyAaKMaemOAaOgabaGaeiikaGIaeGOmaiJaeiykaKcaaOGaei4la8IaemOta40aaSbaaSqaaiabikdaYaqabaGccqGH8aapcqWGobGtdaqhaaWcbaGaemyAaKMaemOAaOgabaGaeiikaGIaeGymaeJaeiykaKcaaOGaei4la8IaemOta40aaSbaaSqaaiabigdaXaqabaaaaa@4178@, then gene *i *is assigned to *G*_1_and gene *j *is assigned to *G*_2_; otherwise, gene *j *is assigned to *G*_1 _and gene *i *is assigned to *G*_2_. In this way, the genes assigned to *G*_1 _tend to be expressed less than those assigned to *G*_2_.

### Data integration

Since rank is invariant to within-array monotonic data transformations, by applying the rank-based TSPG method, which performs on-chip comparisons within each microarray, no data normalization and transformations are required before integration. For microarray data generated from the same platform, we directly merge individual data sets using the common probe sets across all the data sets to form an integrated data set of increased sample size. To merge data from different generations of the same array technology, we first select the common probe sets across the platforms and then merge data using the common probe sets. In that case, a large number of genes, which are not in the common set and may include potential signature genes, will be excluded from analysis. In this study, we focus on integrating microarray data generated using same platform to avoid losing any potential signature genes. Specifically, we integrate microarray data generated from both Affymetrix HuGeneFL and HG-U95A microarray platforms to form two corresponding integrated data sets with large (> 500) sample sizes. These two integrated data sets are used as training data sets to identify a common cancer signature.

### Repeated random sampling and signature gene selection

A recent study by Michiels *et al*. has shown that molecular signatures strongly depend on the selection of patient samples in the training sets and they advocate the use of repeated random sampling for signature validation [23]. Motivated by their work, we combine the TSPG algorithm with a repeated random sampling strategy in order to obtain a more robust and reliable common cancer signature. We randomly select *S*% of the total samples from an integrated training set, with *S *chosen close to 100, specifically *S *= 90, in order to reasonably represent the original training samples. We then apply the TSPG algorithm to the selected subset of the original training set to construct two groups, *G*_1 _and *G*_2_, of genes, with a predefined value of *k*, the number of genes in each group. After repeating this experiment a large number of times, we calculate the appearance frequency of each gene in *G*_*l*_, *l *= 1, 2. A frequency threshold *F *(default, *F *= 80%) is set to select those genes whose appearance frequency exceeds the threshold in *G*_*l*_, *l *= 1, 2. The final common cancer signature consists of all the genes picked from *G*_1 _and *G*_2 _for each of the two integrated training data sets, HuGeneFL and HG-U95A.

### Class prediction

To assess the classification accuracy of the common cancer signature, we build a TSPG classifier based on all the signature genes. The signature genes picked up from *G*_1_'s (respectively, *G*_2_'s) of the two integrated training sets by repeated random sampling form the group *G*_1 _(respectively, *G*_2_) of the TSPG classifier. The classifier votes for class 1 (i.e. normal) if the average relative rank of the genes in *G*_1 _is less than that of the genes in *G*_2_; otherwise, it votes for class 2 (i.e. cancer). We not only use the classifier to predict samples from data sets involved in identifying the signature, but also validate it on independent data sets. A Fisher's exact test is used to assess the significance of the classification accuracy. The classification accuracy, as well as its statistical significance, is reported for all the individual data sets, training and test.

In Table 7 we compared the TSPG classifier with the one described in Rhodes [17], which requires the existence of an ensemble of samples to learn class averages for each gene in the signature. A new sample is then classified according to the class (cancer or normal) which receives the most votes in comparing the new expression values to these two averages, one comparison per gene in the signature. Moreover, these class averages cannot be computed from the same training data from which the signature is induced since the signature is derived from combining multiple signatures derived from different data sets with differing probe sets. Hence, the only choice for testing the Rhodes signature is cross-validation on a test set, which confers a considerable advantage due to the overall homogeneity of the profiles in each test set (e.g., removing any normalization issues), resulting in an upward bias in the classification rates.

## Authors' contributions

LX, under the supervision of RLW and DG, collected the microarray data sets and implemented the algorithms; all three authors developed the methodology and contributed to the final manuscript.
